# The Indigestion of Breast Babies

**Published:** 1897-07

**Authors:** 


### THE INDIGESTION OF BREAST BABIES.

For many reasons less attention has been paid to the gastro-intestinal affections met with in breast babies than in those nursed artificially. Breast milk is the natural and ought to be the sole food of the infant, under physiological conditions, during the first year of life. Unfortunately there is too often a departure from the normal state, and the child, and perhaps also the mother, may suffer during the lactating period. The natural pride and instinct of the mother is apt to lead to the presumption that all is going well with her and the infant, when, perhaps, in reality she is not a good nurse, and the child is suffering more or less, and not thriving.

In America the question of infant feeding in all its aspects has received the well-merited attention which it deserves, and which it has not met with in this country. The greater prevalence of diarrhœal disease during the tropical summer of the American continent has stimulated study and research on this important subject. Milk laboratories have been established in the larger cities, and the feeding of infants has been placed on a comparatively sure and scientific footing. Owing to the researches of such men as Jacobi, Rotch, Holt, Lewis, Smith, Meigs, and others, we are now furnished with scientific data to guide us in the study of the subject. In the author's own country the question of our milk supply is now receiving some attention from sanitarians, but there is as yet no ready means available to the general public of obtaining pure or properly sterilized milk on a large scale, nor of having milk analyzed or tested in laboratories established for the purpose. Whenever the milk of the mother is defective in quantity or quality, the child is apt to suffer. It does not thrive or grow at the normal rate. Instead of being plump and firm and happy, it is soft and flabby, and is always crying, and never appears to be satisfied. Its skin is harsh and dry. The tongue is somewhat red, often slightly furred. Vomiting is not infrequent from gastric catarrh. The stools are unnatural and present various appearances depending on the quality of the milk. They are generally loose, and seldom have the natural mustard color or consistency; but are usually pale, and often of an ashy gray color, sometimes greenish, or mixed gray and green. The soft curd of the mother's milk is present undigested in little granular-looking masses. There is an excess of mucous secretion, sometimes little streaks of blood. As a rule, indigestion of mother's milk is more frequently intestinal than gastric, diarrhœa being more common than vomiting. This appears to be largely due to indigestion of the fatty and proteid elements of the milk. Infants, in regard to their digestive capabilities, are but little men and women, and it is certain they have their idiosyncrasies likewise. The milk of a mother seems to be suited to her own child under physiological conditions.

These two cases are very ordinary ones, and typical of two of the conditions giving rise to indigestion in the infant. Irregular suckling is one of the commonest causes of indigestion in babies. It produces a milk too concentrated, which inevitably causes indigestion in the child. Regulation of the suckling is generally sufficient to give relief. Irregular suckling may be due to two principal causes. It may occur in cases where the milk is normal in quantity and quality, from bad habit on the part of the mother in being over-anxious about her child, and carelessly giving it the breast at irregular times or whenever it cries. The more frequent cause, however, is deficient quantity of milk. In this case the child is unsatisfied and gets the breast too frequently in consequence, with the result that the milk becomes too concentrated and causes indigestion. The remedy is the addition of some substitute feeding.

Inseparably connected with the question of maternal feeding is the no less important one of the artificial rearing of infants who are unable to obtain breast milk. The huge mortality of infants under one year is hardly reduced to a lower level than it was half a century ago, when in England and Wales no less than 76,328 children under twelve months died, out of a total of 350,101 deaths in one year. Want of breast milk and bad artificial feeding are largely responsible for this. Surely it is our duty, as a profession, to try and stem this tide of mortality. There is no way to attain this end but by education—medical education; and let us hope that in the near future we will be in a better position in this respect, and have greater facilities for showing good results in what, it must be admitted, is an important branch of preventive medicine too much neglected.—*Medical News.—Medicine.*

